# Sildenafil Citrate Increases Fetal Weight in a Mouse Model of Fetal Growth Restriction with a Normal Vascular Phenotype

**DOI:** 10.1371/journal.pone.0077748

**Published:** 2013-10-30

**Authors:** Mark Robert Dilworth, Irene Andersson, Lewis James Renshall, Elizabeth Cowley, Philip Baker, Susan Greenwood, Colin Peter Sibley, Mark Wareing

**Affiliations:** 1 Maternal and Fetal Health Research Centre, Institute of Human Development, University of Manchester, Manchester, United Kingdom; 2 Manchester Academic Health Science Centre, St. Mary’s Hospital, Central Manchester University Hospitals NHS Foundation Trust, Manchester, United Kingdom; 3 Faculty of Medicine and Dentistry, University of Alberta, Edmonton, Canada; 4 Liggins Institute, University of Auckland, Auckland, New Zealand; The Ohio State Unversity, United States of America

## Abstract

Fetal growth restriction (FGR) is defined as the inability of a fetus to achieve its genetic growth potential and is associated with a significantly increased risk of morbidity and mortality. Clinically, FGR is diagnosed as a fetus falling below the 5^th^ centile of customised growth charts. Sildenafil citrate (SC, Viagra™), a potent and selective phosphodiesterase-5 inhibitor, corrects *ex vivo* placental vascular dysfunction in FGR, demonstrating potential as a therapy for this condition. However, many FGR cases present without an abnormal vascular phenotype, as assessed by Doppler measures of uterine/umbilical artery blood flow velocity. Thus, we hypothesized that SC would not increase fetal growth in a mouse model of FGR, the placental-specific *Igf2* knockout mouse, which has altered placental exchange capacity but normal placental blood flow. Fetal weights were increased (by 8%) in P0 mice following maternal SC treatment (0.4 mg/ml) via drinking water. There was also a trend towards increased placental weight in treated P0 mice (*P* = 0.056). Additionally, 75% of the P0 fetal weights were below the 5^th^ centile, the criterion used to define human FGR, of the non-treated WT fetal weights; this was reduced to 51% when dams were treated with SC. Umbilical artery and vein blood flow velocity measures confirmed the lack of an abnormal vascular phenotype in the P0 mouse; and were unaffected by SC treatment. ^14^C-methylaminoisobutyric acid transfer (measured to assess effects on placental nutrient transporter activity) per g placenta was unaffected by SC, versus untreated, though total transfer was increased, commensurate with the trend towards larger placentas in this group. These data suggest that SC may improve fetal growth even in the absence of an abnormal placental blood flow, potentially affording use in multiple sub-populations of individuals presenting with FGR.

## Introduction

Fetal growth restriction (FGR) is defined as a baby that fails to reach its genetic growth potential and affects approximately 5–10% of pregnancies worldwide [1]. FGR places a baby at a significantly increased risk of being stillborn whilst babies that survive are more likely to present with childhood morbidities such as cerebral palsy [2]. Additionally, strong correlations exist between low birth weight and an increased risk of adulthood diseases such as hypertension, diabetes mellitus and stroke [3]–[5]. The major cause of FGR is thought to be placental dysfunction [6].

Despite the significant risks associated with FGR-affected pregnancies, there remains no treatment. The only option currently available to clinicians is early delivery of the baby which is itself associated with increased morbidity and/or mortality [7], [8]. Furthermore, there are still no drugs developed specifically for obstetric conditions currently in clinical trials [9]. This has led to the assessment of drugs currently used in clinical practice for other diseases, to be assessed on a re-purpose basis as potential therapeutics in the treatment of FGR.

Sildenafil citrate (SC, Viagra™), initially used as a treatment for pulmonary hypertension and now established as a treatment for erectile dysfunction [10], represents one such potential therapy. SC is a phosphodiesterase 5 (PDE-5) inhibitor, delaying the breakdown of cyclic guanosine monophosphate (cGMP) and enhancing nitric oxide (NO)-dependent vasodilatation. SC has been shown to restore *ex vivo* myometrial artery dilatation from FGR-affected pregnancies [11]. There have now been a number of studies investigating SC as a potential treatment for FGR [12], with a consensus on its effectiveness yet to be reached. SC has been shown to be effective in a mouse model that demonstrates FGR, the catechol-o-methyl transferase (COMT) knockout mouse, where fetal weight was normalized following SC treatment in the final third of pregnancy [13]. In this study, the COMT knockout mouse also demonstrated an abnormal umbilical artery Doppler waveform, a clinical feature in some cases of human FGR, with reduced end diastolic blood flow velocity; this too was normalized following treatment with SC [13]. In a sheep model of FGR induced by maternal nutrient restriction, long-term (from day 28–115 of pregnancy) SC treatment increased both fetal growth and amino acid availability in the conceptus [14]. However, a separate study, also in sheep, in which FGR was induced by ligation of a single uterine artery (causing placental infarction and thus placental insufficiency), demonstrated that acute (24-hour) administration of SC resulted in a decreased uterine artery blood flow [15]. In addition, SC treatment in this study resulted in fetal tachycardia and hypotension, with the authors urging caution regarding the use of SC in pregnancy [15]. In a recent study in which FGR was elicited in the rat by maternal administration of the nitric oxide synthase blocker Nω-Nitro-l-arginine Methyl Ester (L-NAME), it was suggested that SC reduced the number of stillbirth pups but also reduced fetal weight [16]. Despite these concerns, a recent non-randomised clinical trial tested the effectiveness of SC as a treatment in severe early-onset FGR [17] and showed an increase in abdominal circumference of these fetuses compared with an untreated severe early onset FGR group [17].

For these studies listed above, the rationale of SC treatment was that it would improve fetal growth by normalising or increasing uteroplacental blood flow. However, a significant proportion of pregnancies identified as FGR do not present clinically with a vascular phenotype (i.e. abnormal uterine artery or umbilical artery blood flow velocity as assessed by Doppler ultrasound) [18]–[21]. Thus, the question remains as to whether any future SC therapy should only be targeted to those pregnancies with abnormal uterine/umbilical artery Doppler flow-velocity waveforms.

In order to address this question, we employed a mouse model of FGR that does not demonstrate an abnormal uteroplacental vascular phenotype. The model chosen was the placental-specific insulin-like growth factor 2 *(Igf2)* P0 knockout mouse, hereafter referred to as P0. These mice demonstrate a reduction in placental size from embryonic day (E) 12.5, prior to the ensuing FGR first seen at E 16.5 [22], [23]. This timeline suggests that the observed FGR in the P0 knockout mouse is a result of placental insufficiency. Placentas from P0 mice demonstrate abnormal morphology, and are characterized by an increased thickness and reduced surface area of the exchange barrier within the labyrinthine (maternofetal exchange) layer, associated with a decreased permeability to hydrophilic solutes [24]. This abnormal morphology and exchange barrier function of the P0 placenta is similar to that in placentas from FGR-affected human pregnancies [25], [26]. However, *in vitro* myography data suggest that there is no placental vascular defect in these mice [27].

The aim of our study was therefore to test the null hypothesis that SC would fail to increase fetal weight in the P0 knockout mouse because of this lack of an abnormal vascular phenotype.

## Materials and Methods

### Animals

Experiments were performed in accordance with the UK Animals (Scientific Procedures) Act of 1986 under Home Office licence 40/3385. The Local Ethical Review Process of the University of Manchester approved all protocols. When required, anaesthesia was employed to minimise pain and suffering.

Placental specific *Igf*2 (Delta U2 P0) knockout mice, which had deletion of the U2 exon of the *Igf*2 gene, were generated as previously described [28] and were a kind gift from Professor W. Reik and Dr M Constância (Babraham Institute, Cambridge, UK).

Wild type C57Bl/6J female mice (10 to 16 weeks old) and males heterozygous for the P0 deletion (10 weeks to 6 months old) were mated and embryonic day 0.5 of gestation (E 0.5) determined by the discovery of a copulation plug (term = E 19.5). This cross resulted in both WT and P0 pups within a single litter. All animals were provided with nesting material and housed in cages maintained under a constant 12 h light/dark cycle at 21–23°C with free access to food (Beekay Rat and Mouse Diet, Bantin & Kingman, Hull, UK) and water.

On E12.5, animals were randomly assigned into either control or treated groups. Those in the treated group were given 0.4 mg/ml sildenafil citrate (SC; Pfizer, Sandwich, UK), via drinking water, until E18.5 with a fresh bottle made up at E15.5. Control animals remained on standard drinking water. 0.4 mg/ml SC was chosen despite previous studies demonstrating positive effects at a concentration of 0.2 mg/ml [13], [29]. This was because, in the original study [29], the authors were attempting to achieve a dose of SC of 50 mg/kg/d. However, early monitoring of P0 mice suggested average drinking of 3–4 ml of water per mouse per day; 0.2 mg/ml SC dosage would only achieve 24–32 mg/kg/day for a 25 g mouse at E12.5. Hence, a decision was made to increase this dose to 0.4 mg/ml to achieve a therapeutic dose closer to 50 mg/kg/d. This compares with a dose of around 1–2 mg/kg/day previously administered in human pregnancy [17] and reviewed in [12], although caution should be taken when making direct comparisons between species given the faster rate at which SC is metabolized in the mouse versus human [30].

**Figure 1:**
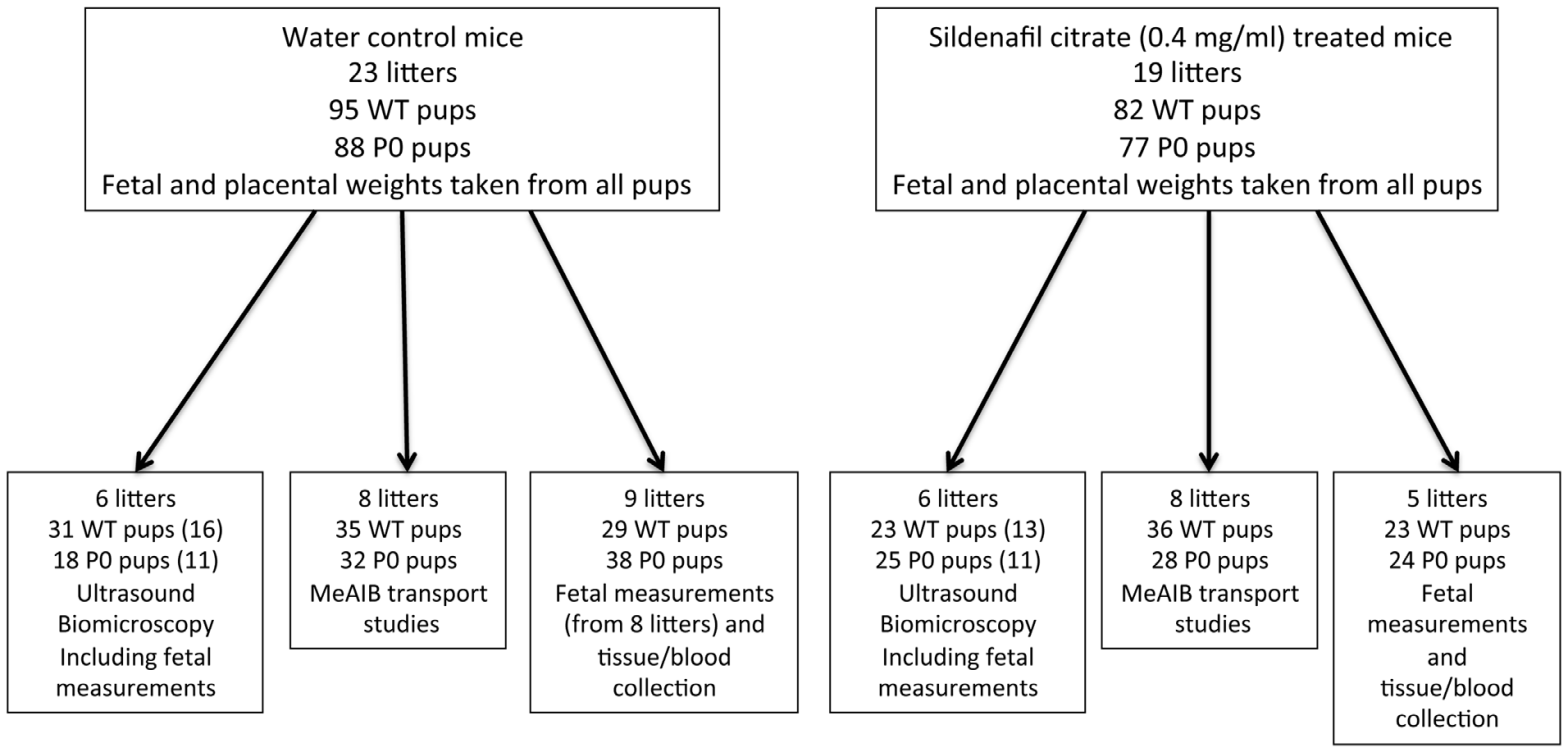
Study design. Number of litters and pups (WT and P0) in each group and each study arm are demonstrated. Sildenafil citrate (0.4 mg/ml in drinking water) was administered from embryonic day (E) 12.5 and all measurements were taken at E 18.5. For ultrasound biomicroscopy, due to necessity to ID pups accurately immediately after scanning (as WT and P0 exist within the same litter), not all pups underwent ultrasound biomicroscopy (numbers in parentheses are those that did.).

In total, 23 control and 19 SC-treated mice were used. From these, mice were either assigned to a group for measurement of umbilical artery/vein blood flow velocity or a group for those undergoing placental clearance/transport studies. Study design can be seen in Figure 1. At E18.5, dams were euthanised and fetuses and placentas were blotted and weighed. In a subset of mice where radioactivity had not been used (14 control and 11 SC-treated mice) fetal anthropomorphic measurements (crown:rump length, abdominal circumference and head circumference) were taken as previously described [31]. Fetal tail tips were collected from all mice for analysis of genotype.

### Fetal Weight Frequency Distribution Curves

Fetal weight histograms were constructed as previously described [32] and a non-linear regression performed (Gaussian distribution). The 5^th^ percentile weight was calculated as: (−Z critical value × SD)+mean

where Z critical value (for the 5^th^ centile) = 1.645 and SD = standard deviation.

### Genotyping

Fetuses were genotyped using genomic DNA extracted from fetal tail tips (DNeasy, Qiagen, Crawley, UK). *Igf*2 P0 mutants were identified via an established PCR method using a primer pair to amplify a 740 bp fragment across the 5 kb deletion (sense, 5′-TCCTGTACCTCCTAACTACCAC -3′; antisense, 5′-GAGCCAGAAGCAAACT -3′) with a third primer amplifying a 495 bp fragment from the WT allele (5′- CAATCTGCTCCTGCCTG-3′), as described previously [33]. PCR conditions are available upon request.

### Umbilical Artery and Vein Blood Flow Velocity

We performed *in vivo* assessment of umbilical blood flow using ultrasound biomicroscopy in order to confirm the lack of a vascular defect in P0 mice. Minimal and maximal blood flow velocities in umbilical artery and vein were assessed at E 18.5 as described previously [13]. Briefly, mice were anaesthetized with isofluorane (3%) in air, with maternal heart rate, respiratory temperature and rectal temperature being recorded throughout. Following hair removal around the abdomen, pre-warmed gel was applied to the skin. Mice were imaged transcutaneously using an ultrasound biomicroscope (Vevo 2100, Visualsonics®, Toronto, Canada) and a 30 MHz transducer operating at 100 frames/s. In color Doppler mode, the high-pass filter was set at 6 Hz and the pulse repetition frequency was set between 4 and 48 kHz. A 0.2 to 0.5 mm pulsed Doppler gate was used, and the angle between the Doppler beam and the vessel was <30°. Images were then recorded for offline analysis. Peak (maximal) systolic velocity (PSV) and end (minimal) diastolic velocity (EDV) were measured from at least 3 cardiac cycles that were not affected by motion caused by maternal breathing and the results were averaged. The pulsatility index (PI) was calculated as PI = (PSV-MDV)/MV where PSV = peak systolic velocity, MDV = minimum diastolic velocity and MV = mean (time-averaged) velocity.

### Unidirectional Maternofetal Clearance of ^14^C-MeAIB Across the Intact Placenta (^MeAIB^ *K*_mf_)

The activity of the placental System A transporter is reduced in human FGR [34], [35] and is first upregulated then reduced at the time FGR is observed in the P0 mouse [22]. We therefore investigated whether SC had any effect on transfer of amino acid via this transporter by measuring the clearance across the placenta of ^14^C-methylaminoisobutyric acid (MeAIB), a non-metabolisable substrate for system A.^ MeAIB^ *K*_mf_ across the intact placenta was measured at E 18.5 using an adaptation of the method of Flexner and Pohl [36] as described previously [23]. Following infusion of ^14^C-MeAIB, exsanguination of the dam occurred between 1 and 5 minutes post-infusion in accordance with previous studies [23], [37]. Fetuses were rapidly collected and assessed for total radiolabel accumulation and compared to a maternal plasma ^14^C-MeAIB disappearance curve (see below).

^MeAIB^ *K*_mf_ (µl/min/g placenta) was calculated as:
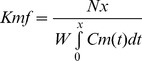
Where, N_x_ = total radiolabel accumulation (dpm) by the fetus (corrected for the fetal tail tip retained for genotyping) at x min after injection of radiolabel into the maternal vein, W = placental wet weight (g) and 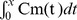 = the time integral of radioisotope concentration in maternal plasma (dpm.min/µl) from 0 to x min (taken from the maternal plasma ^14^C-MeAIB disappearance curve).

### Maternal Plasma ^14^C-MeAIB Disappearance Curve Following Injection into the Maternal Circulation

A maternal plasma ^14^C-MeAIB disappearance curve was constructed from dams at E 18.5 (N = 51) and fitted to a one-phase exponential decay model (r^2^>0.6) as described previously [37]. These 51 dams consisted of the 16 dams in this study together with historical data from C57 dams (crossed with P0 males) from our research group. Disappearance curves were checked to ensure that dams in this study gave comparable values to historical data and that SC did not alter maternal plasma ^14^C-MeAIB disappearance.

### Data and Statistical Analyses

Sample sizes were determined using Altman’s nonogram based upon a 5% significance level and 80% statistical power. All data were normally distributed following a D’Agostino & Pearson normality test and are shown as mean ± SEM. Unless otherwise stated, a Generalized Linear Mixed Models approach, with each litter used as a random effect, was used to assess whether there was a significant effect of genotype and/or treatment. A Sequential Sidak multiple comparisons test was then used to test for differences between groups. *P*<0.05 was deemed statistically significant. Statistical analyses were performed using IBM SPSS Statistics software (IBM, New York, US).

## Results

### Fetal and Placental Weights and Fetal Anthropometric Measurements

**Figure 2:**
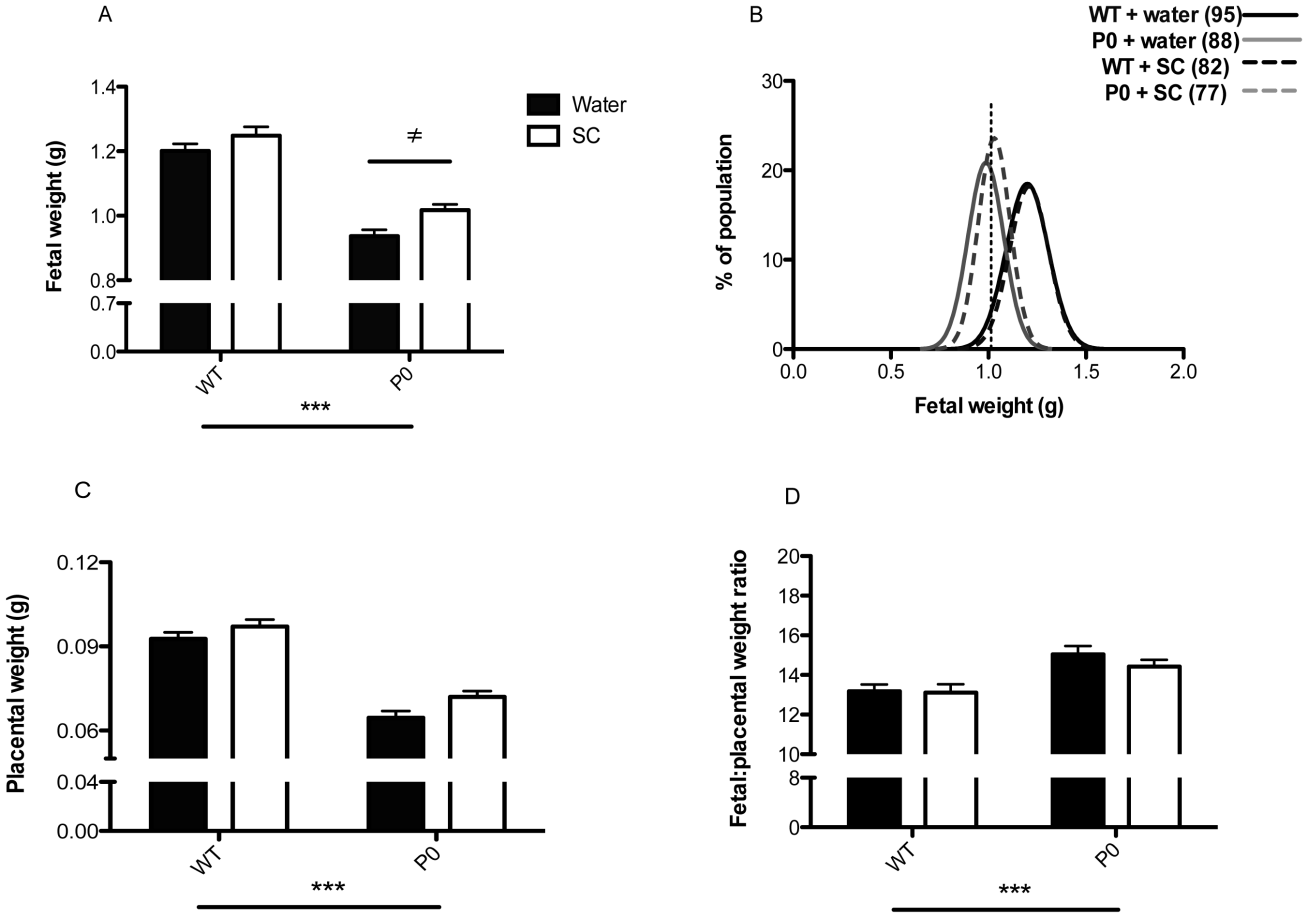
Fetal and placental weights following Sildenafil citrate treatment. Fetal weights (A,B), placental weights (C) and fetal:placental weight ratios (D) at embryonic day 18.5 in wild-type (WT) and placental-specific *Igf2* knockout (P0) mice. Pregnant dams were allowed access to water with (N = 19 litters) or without (N = 23 litters) Sildenafil citrate (SC, 0.4 mg/ml). In figures 2A,C and D, Mean+SEM is shown. Figure 2B demonstrates fetal weights as frequency distribution curves for individual pups; WT control (black solid curve), WT SC (black dashed curve), P0 control (grey solid curve) and P0 SC (grey dashed curve). Vertical dashed line denotes the 5^th^ centile of WT control fetal weights (1.03 g). Individual pup n’s: WT control *n* = 95, WT SC *n* = 82, P0 control *n* = 88, P0 SC *n* = 77. Statistical analyses of genotype and treatment were performed by Generalized Linear Mixed Models test followed by Sequential Sidak multiple comparisons test. ***P<0.001 WT v P0, ^≠^ *P*<0.05 P0 water v P0 SC group.

Litter size was not significantly affected by SC treatment (8.0±0.2 in control versus 8.6±0.3 fetuses per litter in SC, unpaired t test), nor did SC treatment affect the proportion of WT and P0 pups within a litter (52% of pups were WT in both water and SC-treated litters). Fetal and placental weights are shown in Figure 2. Fetal weight (Fig. 2A) was significantly lower in P0 versus WT, independent of treatment (*P*<0.001). Treatment with SC caused an increase in P0 fetal weight (expressed as Mean ± SEM, grams) compared with water-treated P0 mice (1.02±0.02 treated versus 0.94±0.02 untreated, *P*<0.05) with WT weights unaffected by treatment (1.25±0.02 treated versus 1.20±0.03 untreated). Fetal weight frequency distribution curves (Fig. 2B) demonstrated that 49% of P0 fetuses treated with SC were above the 5^th^ centile of the untreated WT weights compared with 25% in the untreated P0 group. Placental weight (Fig. 2C) was decreased in P0 versus WT, which was independent of treatment (*P*<0.001). SC treatment failed to increase placental weight in WT mice (0.097±0.002 treated versus 0.092±0.002 untreated) with P0 placental weight higher but just failing to reach statistical significance at the 5% level following SC treatment (0.072±0.002 treated versus 0.065±0.002 untreated, *P* = 0.056). The fetal:placental (F:P) weight ratio was increased in P0 versus WT, independent of treatment (*P*<0.001) but was not altered following SC treatment in either WT (13.1±0.3 untreated versus 13.2±0.4 treated or P0 (14.4±0.4 treated versus 15.0±0.3 untreated) mice.

Crown:rump length (*P*<0.001), abdominal circumference (*P*<0.001) and head circumference (*P*<0.05) were all significantly reduced in P0 versus WT, independent of treatment (Table 1). SC treatment failed to alter either crown:rump length or head circumference in WT or P0 mice but did increase abdominal circumference in both WT (*P*<0.01) and P0 (*P*<0.05) pups.

**Table 1:** Fetal anthropometric measurements following Sildenafil citrate (SC) treatment.

		WATER (N = 14 litters)		SC TREATED (N = 11 litters)	
		WT (n = 56)	P0 (n = 53)	WT (n = 46)	P0 (n = 49)
**Crown:rump length (mm)**	Genotype***Treatment NS	28.9±0.3	26.6±0.3	29.1±0.4	27.1±0.3
**Abdominal circ (mm)**	Genotype*** Treatment**	26.3±0.3	23.1±0.4	27.2±0.3^a^	24.3±0.5^b^
**Head circ (mm)**	Genotype* Treatment NS	24.8±0.2	24.1±0.2	24.8±0.1	24.6±0.1

Crown:rump length, abdominal and head circumference of WT and P0 fetuses at E18.5 from untreated water (control) and SC treated dams. Mean ± SEM. Statistical analyses were performed by Generalized Linear Mixed Models test followed by Sequential Sidak multiple comparison test. This assessed effects of genotype and treatment.

* *P*<0.05,

** *P*<0.01,

*** *P*<0.001.

a *P*<0.01 versus WT control;

b *P*<0.01 versus P0 control.

### Umbilical Artery and Vein Blood Flow Velocity

**Figure 3:**
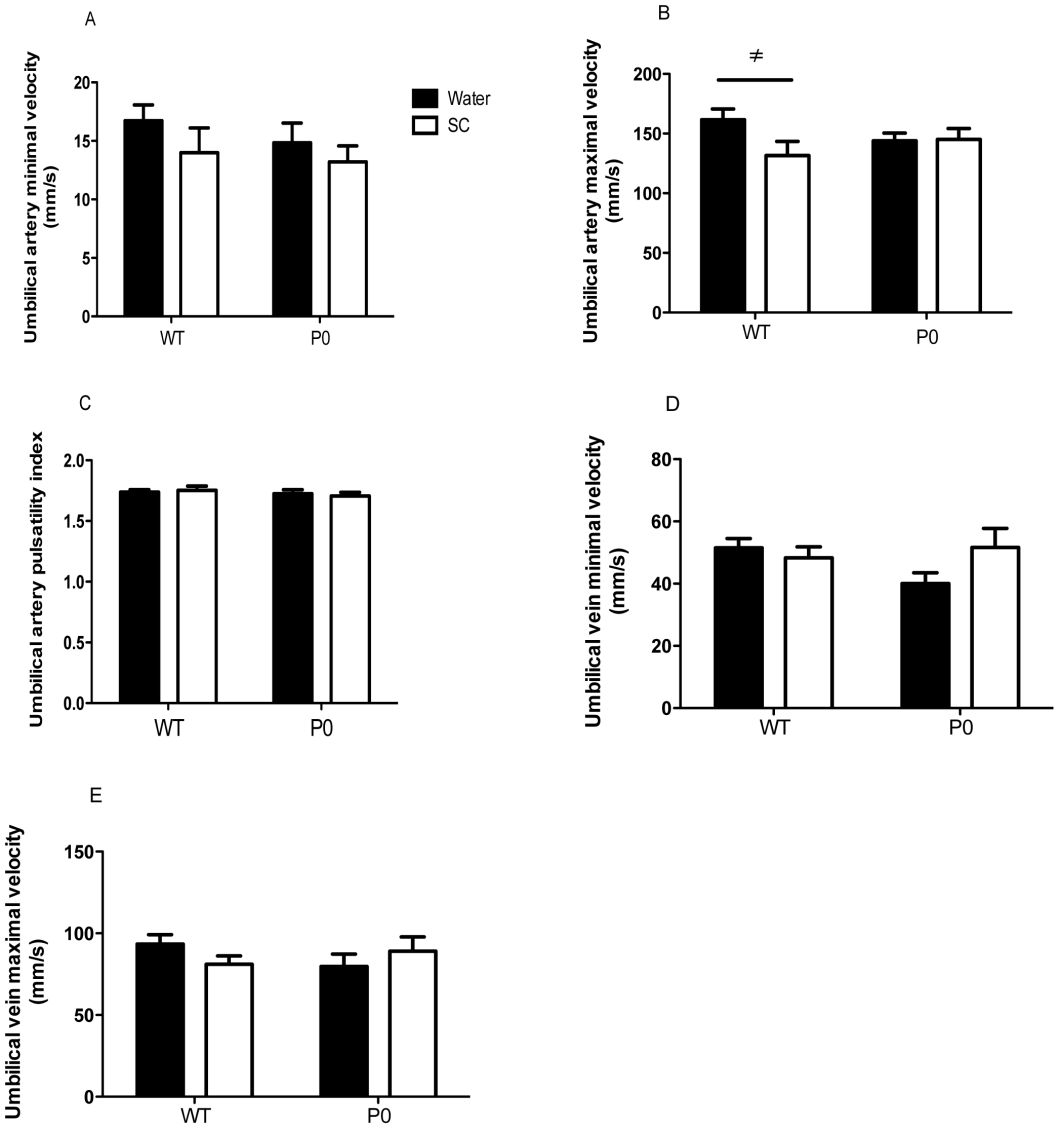
Haemodynamic parameters of umbilical vasculature following ultrasound biomicroscopy. Mean+SEM. Individual pup n’s: WT control *n* = 16, WT SC *n* = 11, P0 control *n* = 11, P0 SC *n* = 13 from 6 untreated and treated litters. Statistical analyses were performed by Generalized Linear Mixed Models test followed by Sequential Sidak multiple comparisons test. This assessed effects of genotype and treatment. ^≠^ *P*<0.05 WT water v WT SC group.

Umbilical artery minimal and maximal velocities and pulsatility index (Fig. 3A–C) and umbilical vein minimal and maximal velocities (Fig. 3D–E) were not significantly different between P0 and WT. Umbilical artery maximal velocity was decreased following SC treatment in WT only (*P*<0.05). All other blood flow measurements were unaffected by SC treatment.

### Unidirectional Maternofetal Clearance of ^14^C-MeAIB Across the Intact Placenta (^MeAIB^ *K*_mf_)

**Figure 4:**
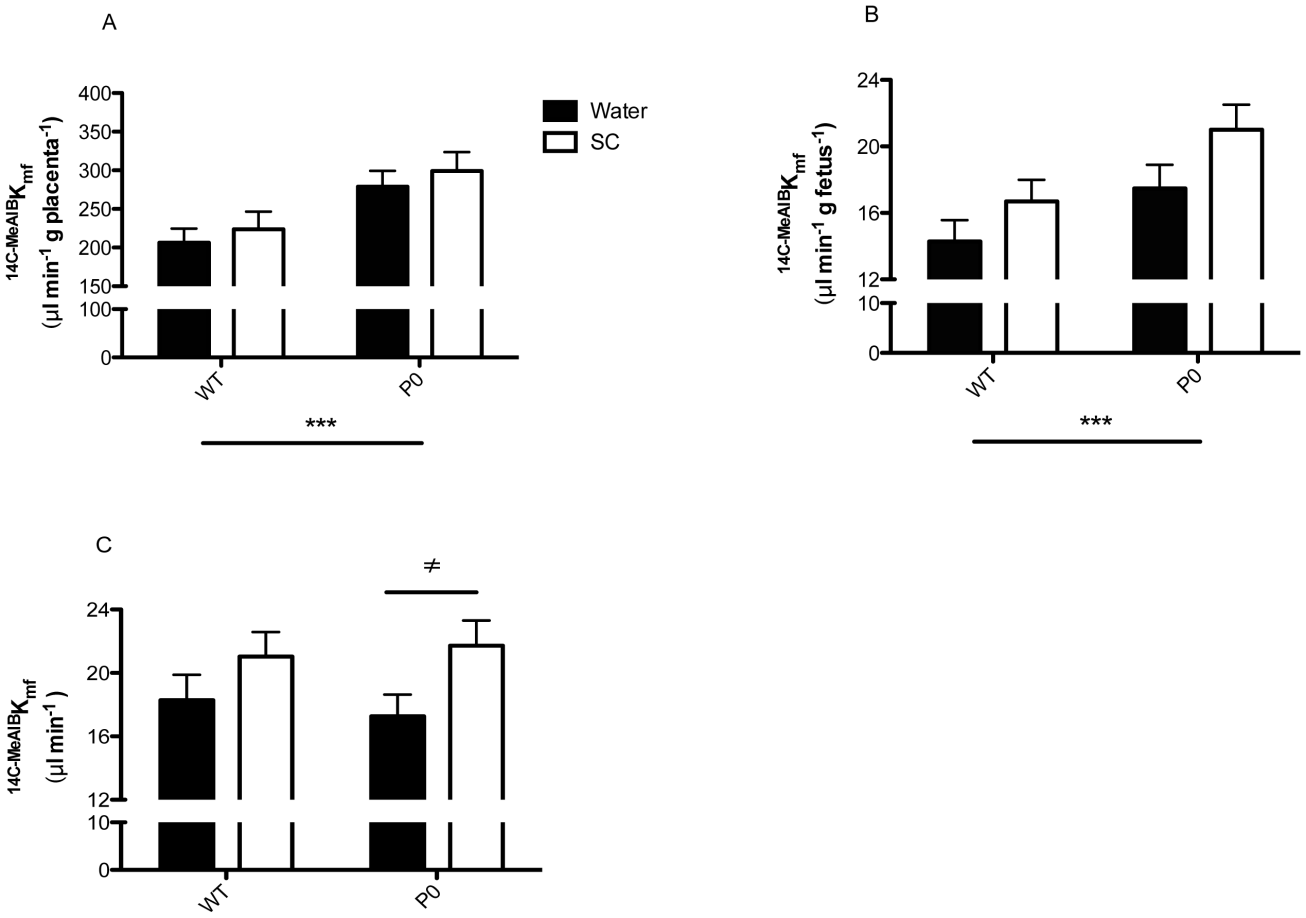
Unidirectional maternofetal clearance of methylaminoisobutyric acid (^MeAIB^ *K*_mf_) following Sildenafil citrate treatment. ^MeAIB^K_mf_ expressed per gram placental weight (measure of placental efficiency, A), per gram fetal weight (measure of appropriate delivery of MeAIB to the fetus, B) and total MeAIB clearance, C. Pregnant dams were allowed access to water with (N = 8 litters, 36 WT and 28 P0 pups) or without (N = 8 litters, 35 WT and 32 P0 pups) Sildenafil citrate (SC, 0.4 mg/ml). Mean+SEM. Statistical analyses of genotype and treatment were performed by Generalized Linear Mixed Models test followed by Sequential Sidak multiple comparisons test. ***P<0.001 WT v P0, ^≠^ *P*<0.05 P0 water v P0 SC group.

^MeAIB^ *K*_mf,_ per gram placenta (Fig. 4A), was significantly higher in P0 versus WT (*P*<0.001) independent of treatment. There was no significant difference between non-treated and SC groups for either WT or P0 mice. When expressed per gram of fetus (Fig. 4B), ^MeAIB^ *K*_mf_ was significantly higher in P0 versus WT (P<0.001) independent of treatment but not significantly different between treatment groups for either WT or P0. When assessed as MeAIB clearance (i.e. per whole placenta, Fig. 4C), there was no significant difference between P0 and WT. However, clearance of ^MeAIB^ *K*_mf_ was significantly higher following SC treatment in P0 mice only (*P*<0.05).

## Discussion

The data presented here demonstrate that, in rejection of the null hypothesis, SC increased fetal growth in FGR-affected (P0) mice. There was also a trend towards increased placental weight in SC-treated versus untreated P0 mice. Whilst the mechanism underlying this apparent increase in P0 placental weight remains unclear, a larger placenta ultimately affords increased nutrient delivery to the fetus and increased fetal weight.

Untreated WT and P0 fetal weights were comparable with previous observations [22]–[24], [32] and confirmed the P0 FGR phenotype in the mice used in this study. Although there was an increase in fetal weight in SC-treated compared with untreated P0 fetuses, the weight of these fetuses remained lower than in WT mice. Thus, SC treatment failed to fully correct fetal weight in FGR mice. However, in order for a treatment for FGR to be clinically relevant, increasing fetal weight above the 5^th^ centile of weights (and certainly above the 10^th^ centile, the small for gestational age threshold) is likely to lead to improved short and long-term outcomes [3], [4], [38]. In this context, SC treatment resulted in 49% of P0 fetuses being above the 5^th^ centile of WT control weights compared with just 25% in untreated P0 mice, again suggesting that the treatment is clinically valuable. Whilst potentially clinically valuable, it should be pointed out that treatment of SC in this model began at E 12.5, prior to the onset of FGR. The fact that the P0 mouse demonstrates a smaller placenta prior to this FGR suggests that, extrapolating these data into the clinic, treatment may be best directed to those pregnancies where a small placenta is apparent. Although accurate estimation of placental size via ultrasound has proven difficult to date, more recent use of 3D power Doppler has shown more encouraging results [39].

Interestingly, SC treatment failed to increase fetal weight in the WT C57Bl/6J pups, in contrast with previous reports in sheep [14], mice [13] and rats [40], [41] which suggest an increased fetal weight in control fetuses from SC-treated mothers.

SC treatment resulted in an increased P0 fetal abdominal circumference, consistent with the increased fetal weight observed in P0 mice. These data accord with previous studies in human [17] and mouse [13] pregnancy which have both reported an increased fetal abdominal circumference in FGR following SC administration. Asymmetric FGR is characterized by a reduced abdominal circumference and presents more commonly as a feature of late-onset FGR, associated with placental insufficiency [21], [42]. We have demonstrated previously that the P0 mouse is a model of late-onset FGR [23], [32] and thus the increase in abdominal circumference following SC treatment shown here may be translated to the human. SC has yet to be tested in this cohort clinically, with the only study thus far on those with severe early onset FGR [17]. It must be emphasized however that in the current study, SC was administered over the final week of pregnancy in mice, roughly equivalent to the end of the second trimester onwards in humans. Thus, therapies targeted to late-onset FGR first need their effectiveness tested over the final 2 or 3 days of gestation in appropriate mouse models. Abdominal circumference was also increased in WT treated mice, despite the lack of an effect on fetal weight. Whilst increasing abdominal circumference in an already appropriately grown fetus may not be a desirable outcome, it is unlikely that SC would be administered prophylactically in the clinic.

Blood flow velocity, estimated by ultrasound biomicroscopy, confirmed the absence of an abnormal vascular phenotype in the P0 knockout mouse, consistent with our previous myography data [27]. The decreased umbilical artery maximal velocity seen in treated versus untreated WT mice was unexpected and is worthy of further investigation, although it is important to note that this difference was not observed in the P0 mouse.

SC only increased placental weight (*P* = 0.056) in the current study in P0 mice, with WT placental weight unaltered. This is in contrast with studies suggesting that SC increased placental weight in control Wistar rats following long-term administration [40], [41] but in agreement with recent work in mice which failed to detect any differences in placental weight following SC treatment [13]. Most other published *in vivo* studies examining the effectiveness of SC in FGR failed to report placental weight, and this does require further investigation.

In order to assess whether the trend towards increased P0 placental size led to subsequent increases in nutrient delivery to the fetus, we assessed placental transport by measurement of the well characterised system A amino acid transport system. System A has been shown to be altered in placentas associated with FGR, both in human [34], [35] and in rodent models [22], [31], [43], [44]. The ^MeAIB^K_mf_, per gram of placenta, was comparable between treated and untreated mice, and so the apparently larger P0 placenta following SC treatment significantly increased total MeAIB delivery to the fetus. This was particularly evident in the SC treated P0 mice. This increased MeAIB delivery affords a potential mechanism for the increased P0 fetal weight observed. This also shows that placental efficiency, in terms of System A transport, was unaffected by SC treatment, a fact supported by the lack of a difference in the fetal:placental weight ratio in the SC treated groups. The mechanism behind the trend towards increased P0 placental weight following SC treatment is currently unknown. Evidence suggests that SC, and other PDE-5 inhibitors such as Taladafil, are able to promote angiogenesis via cGMP in non-placental vascular beds [45]–[47] and future placental vascular casting studies may be useful in this regard. In addition, little is known regarding the effects of SC on trophoblast proliferation. Both of these possibilities are currently being investigated.

This study confirms and extends the evidence that SC treatment may be valuable in FGR. Importantly, the data here show that SC can improve fetal growth in a mouse model of FGR without any detectable placental vascular dysfunction. This is particularly relevant as a significant percentage of FGR cases in the clinic do not demonstrate aberrant uteroplacental blood flow, suggesting that SC may have therapeutic value in a larger than expected population of FGR cases.
